# Medical and Surgical Management of Symptomatic and Asymptomatic Carotid Artery Stenosis: A Comprehensive Literature Review

**DOI:** 10.7759/cureus.43263

**Published:** 2023-08-10

**Authors:** Ahmed B Mohd, Yasmeen Alabdallat, Omar B Mohd, Reem A Ghannam, Seri Sawaqed, Hanan Hasan, Mohamed Ellebedy, Khaled Turkmani, Shakir Al-Ezzi

**Affiliations:** 1 Faculty of Medicine, Hashemite University, Zarqa, JOR; 2 Infectious Disease, Faculty of Medicine, Hashemite University, Zarqa, JOR; 3 Medical Laboratory, The Lab Medical Laboratories, Amman, JOR; 4 Faculty of Medicine, Sohag University, Sohag, EGY; 5 Faculty of Medicine, Syrian Private University, Daraa, SYR; 6 Internal Medicine, Hospital Corporation of America (HCA) Medical City Arlington, Dallas, USA

**Keywords:** best medical therapy, carotid endarterectomy (cea), carotid stenting, carotid artery stenosis, asymptomatic, symptomatic

## Abstract

Carotid artery stenosis is a condition where the carotid artery is blocked by fatty cholesterol deposits called plaque, increasing the risk of stroke. Elderly individuals with high cardiovascular risk are more susceptible, along with smokers, those with high cholesterol, males, and older individuals. Young females may also be affected by fibromuscular dysplasia. Carotid stenosis significantly raises stroke risk, and the severity is closely linked to stroke incidence and other cardiovascular events. Early detection and treatment are essential to prevent complications. Treatment options include medical and surgical interventions, such as carotid endarterectomy (CEA) and carotid artery stenting (CAS). The choice between surgery and medical management varies depending on patient characteristics and risk factors. This review explores carotid artery stenosis pathophysiology, risk factors, the importance of early detection and treatment, and the surgical approaches of CEA and CAS, addressing their roles and controversies. Healthcare professionals must understand these aspects to provide optimal care to patients with this condition.

## Introduction and background

Carotid artery stenosis occurs when the carotid artery becomes obstructed by plaque, which is made up of fatty cholesterol deposits. This condition, known as atherosclerosis, disrupts normal blood flow and increases the risk of stroke [1]. Carotid atherosclerotic plaque is commonly found in elderly patients and those at high cardiovascular risk. When plaque occupies 50% of the carotid lumen, it causes significant narrowing of the artery known as hemodynamically significant carotid stenosis [2].

The prevalence of carotid disease becomes more pronounced with advancing age, affecting approximately 7.5% of men and 5.0% of women above the age of 80 [3]. In addition, carotid artery stenosis can be a result of systemic atherosclerosis. Therefore, any risk factor that predisposes individuals to atherosclerosis, such as smoking, hyperlipidemia, male gender, and age, can also predispose them to carotid artery stenosis. In a small percentage of patients, particularly young females, carotid artery stenosis may be caused by a condition called fibromuscular dysplasia [4].

Carotid disease is associated with one-third of all strokes [4]. The annual stroke rate is 3.3% in individuals with carotid stenosis greater than 75%. Furthermore, patients with severe carotid stenosis have higher rates of cardiac events (8.3%) and death (6.5%) [5]. However, for carotid stenosis less than or equal to 75%, the stroke rate is insignificant at 1.3% annually [5]. These statistics highlight the importance of early detection and treatment of carotid artery stenosis to prevent the development of complications.

Treatment options for carotid stenosis include medical and surgical approaches. Surgical procedures, such as carotid endarterectomy (CEA) and carotid artery stenting (CAS), are available. While both approaches reduce the risk of cerebral ischemia, the decision on whether a patient requires surgical or endovascular therapy or if optimal medical management is sufficient remains controversial [3]. In this review, we delve into the different treatment modalities and emphasize the significance of each surgical approach in managing carotid artery stenosis.

## Review

Symptomatic carotid artery stenosis

Surgical Management

CEA is a commonly performed surgical procedure for treating carotid artery stenosis. During this procedure, the surgeon opens the artery and removes plaque. Research has shown that CEA is beneficial for patients with moderate and severe stenosis, but it does not provide significant benefits for patients with less than 50% stenosis [6]. Emergent CEA (eCEA) carries similar risks as non-eCEA, except for patients who have experienced a transient ischemic attack (TIA), where there may be additional risks [7]. There are two main surgical techniques used for CEA: the classical (conventional) method and the eversion method [8].

In recent years, CAS has emerged as an alternative to CEA, especially for patients under 70 years old, as minimally invasive endovascular techniques have advanced [9]. CAS may be recommended for patients with severe pulmonary disease, recent myocardial infarction (MI), unstable angina, severe congestive heart failure, or other conditions that make them unsuitable for open surgery. It can also be performed in cases where there is recurrent stenosis after a previous endarterectomy, the presence of a tracheostomy, contralateral carotid occlusion, history of contralateral vocal cord damage, or when accessing the narrowed area is challenging with endarterectomy [10].

Transfemoral CAS (TF-CAS) is not advisable for patients with total carotid artery thrombotic occlusion or unfavorable aortic arch anatomy, such as a heavily calcified aortic arch or type 3 aortic arch. It is also not preferred for patients who have had a severe allergic reaction to intravenous contrast dye or have renal impairment [10].

For patients with high-grade carotid artery stenosis, whether symptomatic or asymptomatic, it is recommended to perform carotid interventions (either CAS or CEA) as soon as possible because the highest risk of recurrent stroke occurs within the first seven to 14 days after the onset of symptoms [11]. If the combined risk of stroke and mortality during the perioperative period is less than 6%, CEA can effectively prevent future ischemic events on the same side of the brain in individuals with symptomatic carotid artery disease [12]. CEA has a particularly significant impact on patients with high-grade stenosis, with eight patients needing treatment to prevent one stroke on the same side of the brain over two years. In patients with moderate stenosis and symptoms, 20 individuals would need treatment to prevent one stroke on the same side of the brain over two years [13].

The benefit of CEA decreases as the time from the initial ischemic event increases, especially in women. The treatment benefit is lost in women when surgery is delayed beyond two weeks [14]. Surgeons have historically been cautious about performing surgery within one month after a TIA or minor stroke due to concerns about increased risks during the procedure. However, subgroup analysis has shown that patients who undergo surgery within two weeks of the qualifying event do not have higher risks of stroke and death [14]. Therefore, recent recommendations for secondary stroke prevention advise performing CEA within two weeks for patients who have experienced a TIA or minor stroke [12].

Medical Management

After experiencing a TIA or minor stroke, it is important to promptly evaluate the condition and initiate early preventive treatments, as this can significantly reduce the risk of early recurrence of stroke by approximately 80% [15].

Invasive therapy should be considered for patients with symptomatic severe carotid artery stenosis, unless there are significant risks involved, such as severe cardiopulmonary disease, recent large cerebral infarction, or hemorrhagic conversion. Antiplatelet therapy and intensive management of vascular risk factors should also be initiated immediately, as they play a crucial role in preventing recurrent strokes following carotid revascularization [16].

A study that examined the relationship between blood pressure and stroke risk in patients with carotid stenosis found that most patients can safely lower their blood pressure gradually over a few days. However, individuals with severe bilateral or critical unilateral stenosis should avoid sudden drops in blood pressure and use antihypertensive medications cautiously [17]. In addition, a randomized clinical trial recommended statin therapy even for those with mild or moderate hypercholesterolemia, aiming to reduce low-density lipoprotein cholesterol levels to less than 70 mg/dL [12,18].

Lipid-lowering therapy with statins, with or without ezetimibe, is recommended for the long-term prevention of stroke and cardiovascular events in patients with symptomatic carotid stenosis and atherosclerotic cardiovascular disease [19]. Statins have been shown to significantly reduce overall mortality and the occurrence of fatal/non-fatal strokes [20]. If the target lipid levels cannot be achieved with maximum doses of statins, the addition of ezetimibe (10 mg daily) is considered an alternative [19]. In cases where dyslipidemia or symptomatic carotid stenosis persists despite statin and ezetimibe therapy, PCSK9 inhibitors may be used as an additional or alternative medication [19].

Aspirin is the most commonly prescribed antiplatelet medication and should be initiated promptly. Some evidence suggests that combining aspirin and clopidogrel within 24 hours of a TIA or minor stroke may be more beneficial than using aspirin alone [21]. However, combination therapy is not frequently recommended [22,23].

For patients undergoing CEA, adding clopidogrel to aspirin increases the risk of hemorrhagic complications and may affect hemostasis. Therefore, the safety of combining clopidogrel with aspirin in individuals with symptomatic carotid artery stenosis needs to be established before routinely recommending this combination. Limited evidence exists regarding the use of aspirin and extended-release dipyridamole in patients with acutely symptomatic carotid disease. Oral anticoagulation is not recommended for symptomatic carotid artery stenosis. Due to the possibility of concurrent coronary artery disease, stress testing should be considered even in the absence of angina or other signs of myocardial ischemia [24].

For patients with symptomatic carotid stenosis who are not suitable candidates for carotid interventions, it is recommended to use short-term aspirin with clopidogrel for 21 days, followed by clopidogrel monotherapy, or long-term aspirin with modified-release dipyridamole [19]. If aspirin and clopidogrel cannot be taken for any reason, alternative options include dipyridamole monotherapy or ticagrelor monotherapy [19].

In individuals with multiple vascular risk factors or concurrent symptomatic coronary artery disease, maintaining a target low-density lipoprotein cholesterol level below 70 mg/dL may be preferable. Patients with concurrent symptomatic coronary artery disease, recent coronary stenting, and severe peripheral arterial disease should be prescribed both aspirin and clopidogrel [25].

Asymptomatic carotid artery stenosis

Surgical Management

The decision to administer invasive therapy to individuals with asymptomatic carotid artery stenosis can be challenging. While randomized controlled trials (RCTs) have shown only minimal benefits of CEA in these cases, the two largest RCTs comparing CEA with medical care for asymptomatic individuals demonstrated a slight advantage of surgery in preventing strokes [26]. According to one of these studies, CEA reduced the risk of stroke from 2% per year to 1% per year [27].

It is important to note that the medical treatment arm of these RCTs did not include intensive reductions in blood pressure and cholesterol levels, which are now considered the best forms of medical therapy. It remains uncertain whether reducing the incidence of stroke with current routine medical care can diminish the relative benefit of CEA in individuals with asymptomatic carotid artery stenosis. Patients with a life expectancy of fewer than five years are unlikely to benefit from the modest risk reduction provided by surgery [28]. In addition, patients aged 75 years and older who underwent surgery did not demonstrate benefit due to their higher death rate at follow-up, which was associated with non-cerebrovascular events, such as MI and cancer [28].

Identifying asymptomatic individuals at a higher risk of stroke could enhance the effectiveness of invasive preventive therapy. Increased severity of stenosis [29], progression of stenosis, a history of contralateral symptomatic carotid artery stenosis [28], and higher serum creatinine concentrations are all indicators of an increased risk of ipsilateral ischemic events in asymptomatic patients with carotid artery stenosis [29]. Early data suggest that comprehensive imaging may help identify carotid plaques more likely to cause symptoms, but further research is needed to confirm these findings [16].

Identification of individuals with severe systemic comorbidities for carotid revascularization becomes important with the development of CAS, which is seen as a less invasive therapeutic option. To maximize the minor benefits of CEA in asymptomatic illness, the choice of a surgical team is also essential [30]. According to Walker [27] and Sacco [12], the benefit is only sustained in asymptomatic patients with severe carotid artery stenosis when the perioperative risks of stroke and death remain below 3%.

Prophylactic CEA in patients with asymptomatic carotid stenosis has been shown in randomized studies to moderately reduce the incidence of stroke compared to medical therapy alone, provided that the procedure's morbidity and mortality rates do not exceed 3% [31,32].

Despite criticisms questioning the modest benefit of surgery and its cost-effectiveness, these data provide "evidence-supported" motivation for performing CEA more frequently on patients with asymptomatic carotid stenosis. Moreover, these studies were conducted in the 1980s to the early 2000s when the only available antiplatelet treatment was aspirin and before the advent of statins, angiotensin-converting enzyme inhibitors, and novel antihypertensive medications [33].

Best Medical Treatment (BMT)

The implementation of best medical treatment (BMT) is considered the fundamental approach in managing patients with both asymptomatic and symptomatic carotid artery stenosis. BMT includes various interventions, such as the use of statins for dyslipidemia, smoking cessation, weight management, glycemic control in diabetic patients, hypertension management, antiplatelet therapy (e.g., low-dose aspirin), and promoting an overall healthy lifestyle [34-37].

The latest guideline from the European Society for Vascular Surgery (ESVS) recommends behavioral counseling regarding a healthy diet, smoking cessation, physical activity, and antihypertensive treatment for patients with asymptomatic and symptomatic carotid disease [19].

Regarding the use of aspirin, an RCT found no significant difference in the occurrence of TIA, ischemic stroke, unstable angina, MI, and cardiovascular-related deaths between the placebo and aspirin groups [38]. However, an observational study reported that aspirin significantly reduced the risk of ipsilateral stroke or TIA and any stroke or death due to cardiovascular events [39].

For diabetic patients with acute coronary syndrome (ACS), optimal glycemic control is recommended [19]. In addition, intensive blood pressure control in diabetic patients has been associated with a 44% lower relative risk of stroke compared to patients with higher blood pressure [40]. For ACS patients with diabetes, a blood pressure goal of less than 140/85 mmHg is recommended [41].

BMT is an effective management strategy for most patients. However, a small proportion of patients may require additional procedures to prevent stroke [42]. BMT is typically applied when a patient has low-grade stenosis (<50%), and for patients with stenosis grade 50-69%, yearly follow-up is recommended, but the benefit of revascularization is still uncertain [43]. When a patient has a stenosis grade of ≥70%, surgical procedures are considered a possibility [43-46].

In an RCT (SPACE-2) comparing three interventions, i.e., CEA with BMT, CAS with BMT, and BMT alone, on patients with asymptomatic carotid artery stenosis, the BMT group showed the best physical activity outcomes. They also achieved better risk factor modification, and no deaths were reported, while the death rates were 2.0% and 2.5% for CEA and CAS, respectively [47,48]. However, the study's sample size was insufficient, and therefore, the RCT did not provide definitive results.

**Figure 1:**
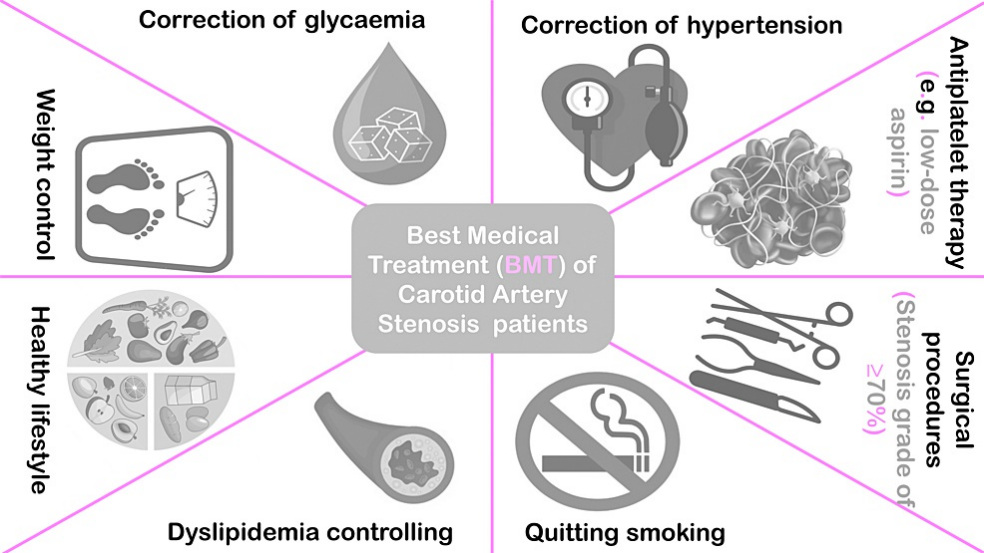
BMT for carotid artery stenosis. BMT: best medical treatment Image Credits: Hanan Hasan

**Figure 2:**
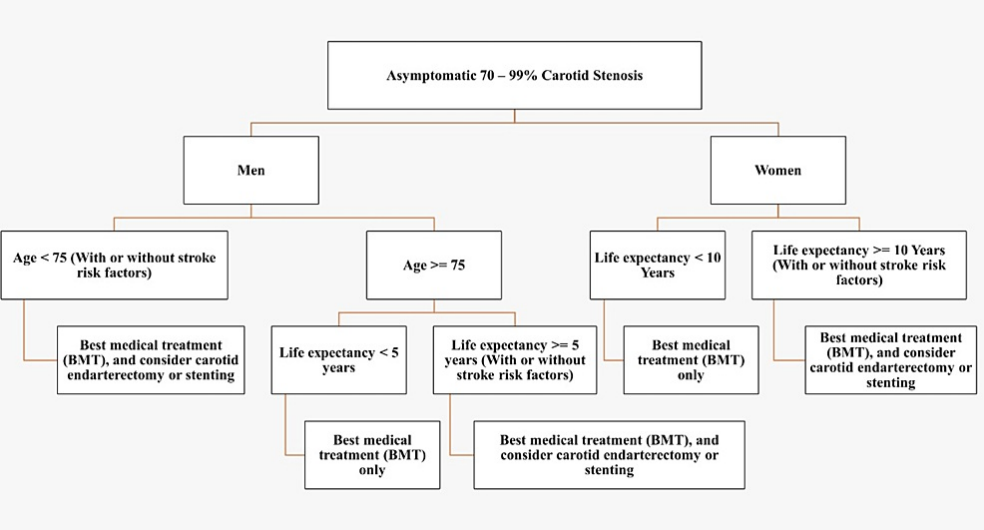
Different approaches to the management of asymptomatic carotid artery stenosis in men and women. Image Credits: Yasmeen Alabdallat

The combined periprocedural stroke and death rate was 2.0% after CEA and 2.5% after CAS, but there were no fatalities or strokes among patients assigned to BMT within 30 days. Although the results are encouraging, it will need further research to elucidate the ambiguities in this crucial area because of the broad confidence ranges caused by the small sample size. Long-term results have not yet been received [47]. In fact, it is debatable if advancements in BMT have eliminated the requirement for carotid surgeries [49]. Figure 1 demonstrates the BMT for carotid artery stenosis. Figure 2 demonstrates the different approaches to the management of asymptomatic carotid artery stenosis in men and women.

Surgical methods of treatment

Several RCTs have compared CEA and CAS and have shown similar outcomes in terms of postoperative complications, including restenosis, the need for revascularization, MI, long-term stroke, and death [50]. Despite numerous studies based on recent RCTs comparing CAS and CEA, it remains unclear which method is superior.

CAS is associated with a lower risk of MI, cranial nerve injuries, access site hematoma, and other non-stroke complications, but it carries a higher risk of cerebrovascular accidents compared to CEA [51-53]. CAS also has a slightly higher risk of periprocedural stroke or death in asymptomatic patients and an even higher risk in symptomatic patients, especially in individuals over 70 years old [53]. In addition, CAS tends to be more effective in younger patients, while CEA may be more beneficial for older patients. Symptomatic patients, regardless of the treatment method, have a higher incidence of stroke [51]. Generally, CAS is as effective as CEA in preventing recurrent stroke during the periprocedural period, but CEA is considered safer and more effective in the long term [53]. For asymptomatic patients undergoing carotid revascularization, whether CEA or CAS, there is no difference in complications between both sexes. However, symptomatic women may have an increased risk of postoperative complications after both CEA and CAS [54].

In cases where redo CEA is performed following prior ipsilateral CEA, higher mortality rates have been observed compared to redo CAS. However, the rates of stroke and MI are similar between the two procedures. Therefore, it is recommended to avoid redoing CEA or performing it under regional anesthesia. Conversely, redo CAS and CEA have similar outcomes in terms of stroke, death, and surgery-related complications following prior CAS. However, redo CEA is associated with an increased risk of stroke and death compared to primary CEA among asymptomatic patients, but no significant difference was observed among symptomatic patients [55,56]. Hence, it is also recommended to avoid CEA following prior ipsilateral CAS, especially in asymptomatic patients with serious systemic disease [57,58].

Transcarotid artery revascularization (TCAR) is a less invasive surgical treatment option for patients with carotid stenosis. It involves a smaller incision than traditional CEA and is significantly associated with a lower risk of stroke or death compared to TF-CAS [59]. Transcarotid stenting has shown similar outcomes to CEA but with fewer cranial nerve injuries [60]. TCAR is associated with a significantly lower risk of in-hospital stroke/death, stroke, stroke/TIA, MI, and stroke/death/MI compared to redo-CEA. In addition, TCAR carries a lower risk of in-hospital stroke/TIA compared to TF-CAS [61]. Risk factors, such as previous stroke, ischemic heart disease (IHD), and the degree of stenosis, have been found to be associated with postoperative complications. Having this information can help predict the risk of postoperative complications [62]. Patients undergoing carotid revascularization with a history of radiation therapy to the neck are at higher risk of developing temporary cranial nerve injury during CEA and also at increased risk of late cerebrovascular events and re-stenosis following CAS [63].

However, there is no clinical benefit to intraoperative shunting during CEA, even in patients who may be at high risk for intraoperative cerebral hypoperfusion due to severe stenosis or occlusion of the contralateral carotid artery [64]. There are two types of intraoperative shunting during CEA: routine shunting and selective shunting. However, Aburahma in his study about shunting during CEA has demonstrated that shunting is connected to a low stroke rate [65]. 

Risk modeling with the inclusion of brain and carotid plaque imaging to select patients for interventions

One of the leading causes of an ischemic stroke is thromboembolism brought on by carotid plaque. Numerous RCTs have shown that CEA is beneficial as a preventive measure in those with high-grade carotid stenosis [27,66]. CAS has lately become more popular as an alternative to CEA [67]. Although CAS is a less invasive procedure, one of its disadvantages is the considerably high incidence of distal emboli, although most are subclinical [68]. Therefore, the degree of carotid stenosis and symptoms have been used as the basis for CEA and CAS indications.

Recent histopathologic investigations have shown growing evidence of “vulnerable plaque,” which is defined by the presence of a lipid-rich necrotic core, burst fibrous cap, and intraplaque bleeding, each of which is connected to an elevated risk for thromboembolic events leading to ischemia [69,70]. Alternative criteria to recognize high-risk plaque characteristics may enhance risk assessment and enable focused action. Furthermore, it has been observed that carotid plaque magnetic resonance imaging (MRI) results correspond with histological results in identifying the major carotid lesions’ components, such as the lipid-rich necrotic core [71-73], intraplaque bleeding [72-74], and ruptured fibrous cap [75].

According to some studies, the presence of vulnerable characteristics, such as intraplaque hemorrhage, which was seen as hyperintense signal intensity by T1-weighted carotid MRI, was linked to a higher risk of ischemic events in the future during clinical follow-up [76-79]. On T1-weighted images, acute intraplaque hemorrhage was histologically distinguished by intact red blood cells (RBCs) with intracellular methemoglobin, and it looked iso-to hypointense on T2-weighted images [80,81].

Moreover, numerous investigations have shown that intraplaque hemorrhage, which preoperative carotid MRI identifies, might forecast particle embolization during the dissection phase of CEA or following the treatment process [76,82].

Consequently, constructing a risk-prediction algorithm may be made possible by MRI of carotid plaque, particularly T1- and T2-weighted images. In addition, nomograms, statistically based tools that present the overall likelihood of a particular result for a given patient, can be used to represent prediction models visually [83].

Medical versus surgical prognosis

A study conducted by Bonati et al. compared the outcomes of endovascular therapy and conventional carotid surgery in patients with carotid stenosis. The study found that within one month of treatment, the rates of major outcomes, such as incapacitating stroke or death and any stroke lasting more than seven days or resulting in death, did not differ significantly between the two treatment approaches. However, cranial neuropathy occurred more frequently in patients who underwent surgery, and major groin or neck hematoma occurred more frequently after surgery compared to endovascular treatment. After one year, severe ipsilateral carotid stenosis was more common in the endovascular treatment group. However, a survival study up to three years after randomization showed no substantial difference in the risk of ipsilateral stroke between the two groups [44].

In another study comparing CAS with CEA, the incidence of stroke, death, or procedural MI was higher in the stenting group compared to the endarterectomy group. The risks of any stroke and all-cause death were also higher in the stenting group. There were fewer cases of procedural MI in the stenting group, but all of them were fatal. Cranial nerve palsy occurred less frequently in the stenting group compared to the endarterectomy group, and there were fewer hematomas in the stenting group [31].

The indications for CEA and CAS in the treatment of cervical carotid stenosis are not yet fully established. However, Tsukahara conducted a study suggesting that by selecting appropriate surgical methods based on the characteristics of carotid plaque and other sclerotic lesions in each patient, CEA and/or CAS can be used to treat stenotic carotid lesions with relatively low morbidity and mortality rates, even in patients with high medical risks or bilateral carotid stenosis [32].

In most people with ACS, medical therapy is currently as effective as CEA. However, current estimates indicate that only five percent of ACS patients benefit from CEA in the era of contemporary pharmacological polytherapy, which includes antiplatelets, statins, and angiotensin-converting enzyme inhibitors [84].

Using vascular diameters to predict surgical outcomes

Yan et al. conducted a retrospective analysis on 31 patients who underwent recanalization surgery for internal carotid artery (ICA) occlusion [85]. The patients were examined using high-resolution vessel wall MRI (HRVWI) with contrast. The occlusion levels were classified according to Bouthillier's ICA classification, which divides the ICA into seven segments: C1 (cervical), C2 (petrous), C3 (lacerum), C4 (cavernous), C5 (clinoidal), C6 (ophthalmic), and C7 (communicating).

The study found that the mean diameter ratio (insulin-to-carbohydrate ratio (I/C ratio)) of the proximal occlusive part of the ICA, comparing the ipsilateral side to the contralateral side, was strongly associated with the outcome of the recanalization surgery. Analyzing each individual segment, the study revealed that the I/C ratios of C1 and C2 were positively correlated with the success of the recanalization procedure. Furthermore, the diameter of the C7 segment showed a positive correlation.

The significance of this study lies in the development of a model that can provide valuable information for identifying suitable candidates for recanalization surgery. By considering the I/C ratios and segment-specific data, the model can help discriminate patients who are eligible for this surgical intervention [85].

Neurocognitive improvements after stenting

**Figure 3:**
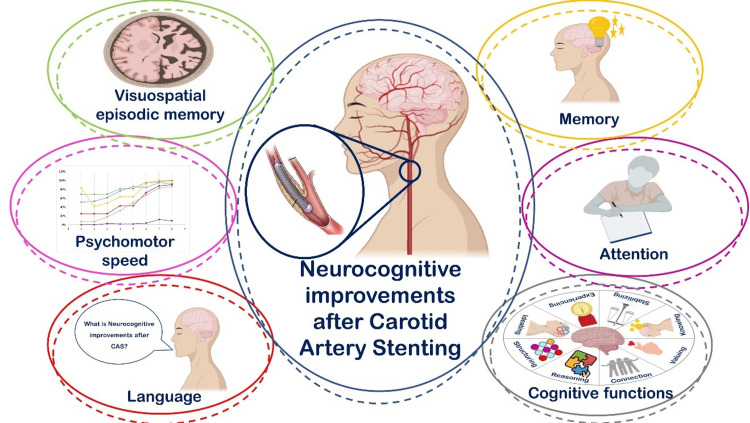
Neurocognitive improvements following carotid artery stenting. Image Credits: Hanan Hasan

Studies show that the benefit of CAS does not only rely on reducing the risk of stroke, it also improves the cognitive function of the brain, as shown in Figure 3 [86,87]. In a study including 47 patients to assess the psychomotor speed, visuospatial episodic memory, executive function, and verbal fluency of patients, all of the aspects except verbal fluency have shown improvement [88]. Furthermore, in a study including 31 patients, the patients showed improvements in attention, memory, visuospatial, and language. However, as in a previous study, there was no improvement in fluency [89]. Meanwhile, in a study including 17 patients, the patients showed no improvement in psychomotor speed [90]. In addition, one study mentioned that CAS does not differ much from carotid artery endarterectomy in improving cognitive functions [86]. 

## Conclusions

A severe medical issue called carotid artery stenosis is characterized by carotid artery occlusion, which raises the risk of stroke. Carotid artery stenosis is more common in the elderly and in people at high cardiovascular risk, and it is influenced by risk factors, including smoking, hyperlipidemia, male gender, and age.

Both medical and surgical methods can be used to treat carotid artery stenosis. When choosing a course of medical or surgical therapy, it is important to carefully assess the unique patient features and risk factors. To offer the best care and enhance patient outcomes, healthcare practitioners should work to have a thorough awareness of carotid artery stenosis and its treatment options.
